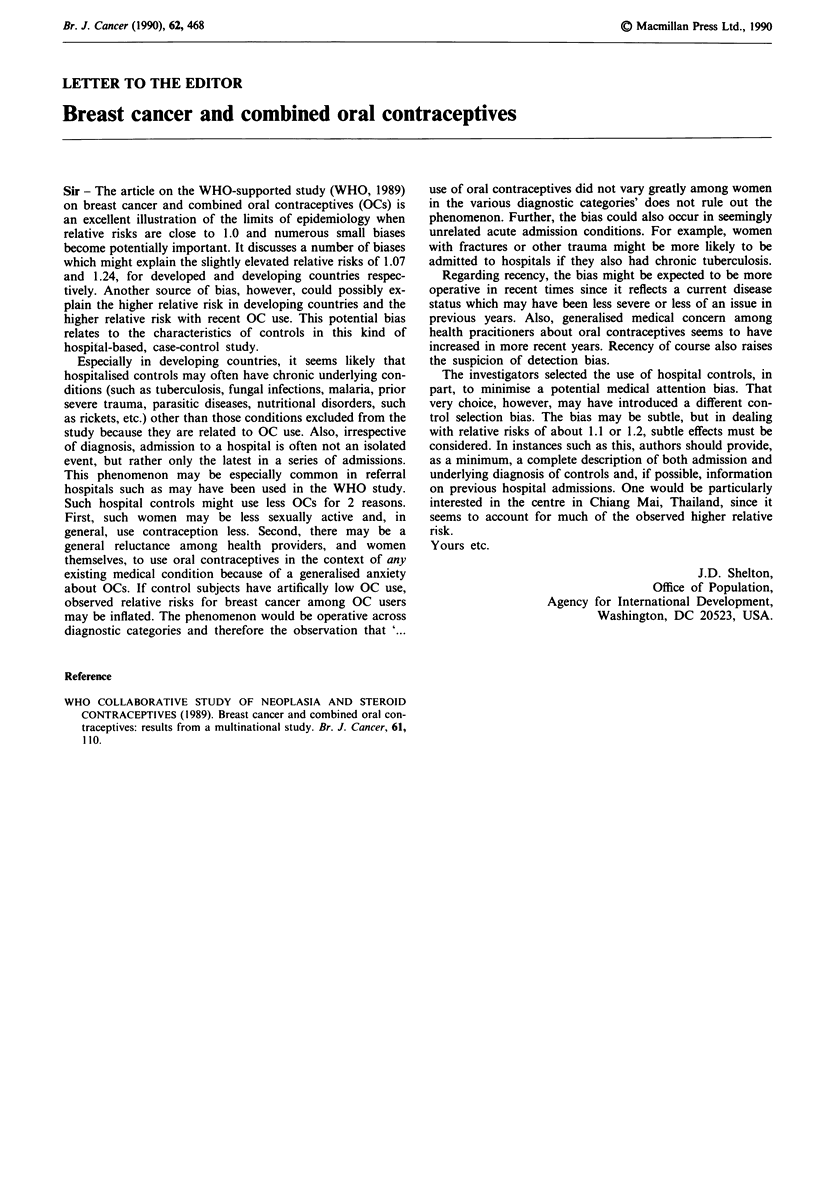# Breast cancer and combined oral contraceptives.

**DOI:** 10.1038/bjc.1990.321

**Published:** 1990-09

**Authors:** J. D. Shelton


*Br. J. Cancer* (1990), **62**, 468 © Macmillan Press Ltd., 1990

### LETTER TO THE EDITOR

### Breast cancer and combined oral contraceptives

**Sir** – The article on the WHO-supported study (WHO, 1989) on breast cancer and combined oral contraceptives (OCs) is an excellent illustration of the limits of epidemiology when relative risks are close to 1.0 and numerous small biases become potentially important. It discusses a number of biases which might explain the slightly elevated relative risks of 1.07 and 1.24, for developed and developing countries respectively. Another source of bias, however, could possibly explain the higher relative risk in developing countries and the higher relative risk with recent OC use. This potential bias relates to the characteristics of controls in this kind of hospital-based, case-control study.

Especially in developing countries, it seems likely that hospitalised controls may often have chronic underlying conditions (such as tuberculosis, fungal infections, malaria, prior severe trauma, parasitic diseases, nutritional disorders, such as rickets, etc.) other than those conditions excluded from the study because they are related to OC use. Also, irrespective of diagnosis, admission to a hospital is often not an isolated event, but rather only the latest in a series of admissions. This phenomenon may be especially common in referral hospitals such as may have been used in the WHO study. Such hospital controls might use less OCs for 2 reasons. First, such women may be less sexually active and, in general, use contraception less. Second, there may be a general reluctance among health providers, and women themselves, to use oral contraceptives in the context of *any* existing medical condition because of a generalised anxiety about OCs. If control subjects have artifically low OC use, observed relative risks for breast cancer among OC users may be inflated. The phenomenon would be operative across diagnostic categories and therefore the observation that '... use of oral contraceptives did not vary greatly among women in the various diagnostic categories' does not rule out the phenomenon. Further, the bias could also occur in seemingly unrelated acute admission conditions. For example, women with fractures or other trauma might be more likely to be admitted to hospitals if they also had chronic tuberculosis.

Regarding recency, the bias might be expected to be more operative in recent times since it reflects a current disease status which may have been less severe or less of an issue in previous years. Also, generalised medical concern among health pracitioners about oral contraceptives seems to have increased in more recent years. Recency of course also raises the suspicion of detection bias.

The investigators selected the use of hospital controls, in part, to minimise a potential medical attention bias. That very choice, however, may have introduced a different control selection bias. The bias may be subtle, but in dealing with relative risks of about 1.1 or 1.2, subtle effects must be considered. In instances such as this, authors should provide, as a minimum, a complete description of both admission and underlying diagnosis of controls and, if possible, information on previous hospital admissions. One would be particularly interested in the centre in Chiang Mai, Thailand, since it seems to account for much of the observed higher relative risk.

Yours etc.

J.D. Shelton,
Office of Population,
Agency for International Development,
Washington, DC 20523, USA.

### Reference

WHO COLLABORATIVE STUDY OF NEOPLASIA AND STEROID CONTRACEPTIVES (1989). Breast cancer and combined oral contraceptives: results from a multinational study. *Br. J. Cancer*, **61**, 110.